# *Escherichia coli* protein synthesis is limited by mRNA availability rather than ribosomal capacity during phosphate starvation

**DOI:** 10.3389/fmicb.2022.989818

**Published:** 2022-12-22

**Authors:** Rocio Espinosa, Michael Askvad Sørensen, Sine Lo Svenningsen

**Affiliations:** Section for Biomolecular Sciences, Department of Biology, University of Copenhagen, Copenhagen, Denmark

**Keywords:** phosphate starvation, protein synthesis regulation, macromolecular synthesis, resource allocation, rRNA stability, bacterial stress response, *Escherichia coli*

## Abstract

Protein synthesis is the most energetically costly process in the cell. Consequently, it is a tightly regulated process, and regulation of the resources allocated to the protein synthesis machinery is at the heart of bacterial growth optimization theory. However, the molecular mechanisms that result in dynamic downregulation of protein synthesis in response to nutrient starvation are not well described. Here, we first quantify the *Escherichia coli* response to phosphate starvation at the level of accumulation rates for protein, RNA and DNA. *Escherichia coli* maintains a low level of protein synthesis for hours after the removal of phosphate while the RNA contents decrease, primarily as a consequence of ribosomal RNA degradation combined with a reduced RNA synthesis rate. To understand the molecular basis for the low protein synthesis rate of phosphate-starved cells, template mRNA for translation was overproduced in the form of a highly induced long-lived mRNA. Remarkably, starved cells increased the rate of protein synthesis and reduced the rate of ribosomal RNA degradation upon mRNA induction. These observations suggest that protein synthesis in phosphate-starved cells is primarily limited by the availability of template, and does not operate at the maximum capacity of the ribosomes. We suggest that mRNA limitation is an adaptive response to phosphate starvation that prevents the deleterious consequences of overcommitting resources to protein synthesis. Moreover, our results support the model that degradation of ribosomal RNA occurs as a consequence of the availability of idle ribosomal subunits.

## 1. Introduction

Phosphorus (P) is an integral and irreplaceable element for living cells and is regarded as a primary geochemically limiting resource for biological mass production on Earth. In *Escherichia coli*, as is typical for living cells, phosphorus is mainly assimilated as orthophosphate (Pi) during the synthesis of adenosine triphosphate (ATP; reviewed in Wanner, 1996). More than half of the total P of a cell is found in RNA, and the remainder is distributed in phospholipids, DNA, low-molecular-mass metabolites such as nucleotides and sugar-phosphates, and inorganic polyphosphate (Wade, 1952). Although proteins do not contain appreciable amounts of phosphate, protein synthesis is directly dependent on the phosphate-rich ribosomal RNA (rRNA), transfer RNA (tRNA), messenger RNA (mRNA), ATP and GTP. Cells that starve for phosphate are therefore compromised in synthesis of the major macromolecules RNA, DNA, and protein.

Protein synthesis is the most energetically demanding process in fast-growing cells, and the translational machinery accounts for a large and adjustable fraction of cellular mass. The deduced bacterial “growth laws” for cultures in balanced growth suggest that *E. coli* reaches a maximal growth rate for a given nutritional quality of the growth medium through near-optimal resource allocation by proteome partitioning. The fraction of the proteome that is dedicated to protein synthesis machinery, and even the sub-partitioning within this fraction (ribosomal proteins, elongation factors, amino-acyl tRNA synthetases and ribosome-affiliated proteins), show a clear growth-rate dependence and the control of its size is at the core of resource allocation optimization theory (Scott et al., 2010; Klumpp et al., 2013; Scott et al., 2014). These models and growth laws are, however, restricted to describe the balanced growth situation where *E. coli* grows at a constant exponential rate.

Outside of the steady state growth regime much less is understood about the prioritization made by *E. coli* regarding the resources allocated to protein synthesis. In a recent study using the slow-growth steady-state approximation of chemostat cultures that grow at a constant rate under limitation for an essential nutrient, it was shown that limitations on carbon (C), nitrogen (N) or phosphorus (P) at identical slow growth rates triggered distinct adaptations of the protein synthesis machinery (Li et al., 2018). During growth in N- or C-limited media, *E. coli* maintained similar ribosome levels as judged by the RNA/protein ratio, but the ribosomes of N-limited cells operated at reduced elongation rates, while C-limited cells increased the fraction of ribosomes that were inactive and free of mRNA, and thus reduced the number of active ribosomes. When P was the limiting nutrient, the RNA/protein ratio was markedly lower than for N- or C-limited cultures growing at the same rate, suggesting the P-limited cells obtained the growth rate despite harboring fewer ribosomes (Li et al., 2018).

We have shown that steady-state cultures exposed to abrupt and complete starvation for C, N, or P, not only limit ribosome synthesis, but also dynamically down-regulate their ribosome content through degradation of rRNA (Fessler et al., 2020; Himeoka et al., 2022; Prossliner et al., 2022). This agrees with previous reports using varying starvation conditions (Davis et al., 1986; Zundel et al., 2009; Basturea et al., 2011; Piir et al., 2011; Sulthana et al., 2016). We found that starvation for P resulted in the fastest degradation kinetics among the three conditions and in agreement with the chemostat experiments, P-starved populations reached the lowest content of full-length rRNA.

At present, it is not well understood how cells regulate the extent of rRNA degradation to adapt to different nutrient-limited growth conditions. In general, only a small number of regulatory factors are known to regulate the levels of the protein synthesis machinery. Most dominantly, the alarmones guanosine tetra- and penta-phosphate, commonly referred to jointly as (p)ppGpp, trigger the stringent response, a coordinated response to many sources of stress in the cell, including nutrient starvation (Cashel and Gallant, 1969). In *E. coli*, (p)ppGpp binds to RNA polymerase to regulate transcription of rRNA, tRNA and ribosomal protein genes, as well as up- and downregulation of expression of other genes, such as those involved in amino acid biosynthesis and GTP metabolism, respectively (Wang et al., 2020). While (p)ppGpp is the key factor responsible for the stark reduction of rRNA and tRNA *synthesis rates* upon nutrient starvation, including P starvation, it is not known to be involved in regulating rRNA *degradation rates*. The specific response to P limitation or depletion is regulated by the two-component system PhoR-PhoB. PhoB is a response regulator, which is activated when the histidine kinase PhoR detects P limitation. In response, PhoB changes the transcriptional profile of the cell by regulating the expression of genes that contain an upstream Pho box (Blanco et al., 2002). The central response revolves around the activation of the high-affinity Pi transport system encoded by *pstSCAB*, and the alkaline phosphatase PhoU (reviewed in Gardner and McCleary, 2019). In essence, the regulatory response to P depletion thus consists in part of halted synthesis of RNA, in particular rRNA and tRNA, and in part of production of P-scavenging proteins. How the high rate of rRNA degradation seen under P starvation is coordinated with these other parts of the starvation response is not yet clear.

Here, we first measure changes in the levels of RNA, DNA and protein that occur upon P starvation in *E. coli* to characterize the resource-allocation-level response to this specific type of nutrient starvation. Secondly, we induce a change in the size of the mRNA pool by means of an overexpressed, long-lived hybrid mRNA, and show that P-starved cells have the capacity to synthesize more protein in the presence of the long-lived hybrid mRNA, suggesting that ribosomes are not *per se* limiting for translation under P starvation. Rather, our results suggest that the limiting factor for translation is the availability of mRNA. Thirdly, rRNA degradation is less severe in the presence of the long-lived hybrid mRNA. Our results support the model that the degree of rRNA degradation upon starvation is regulated by the protein synthesis activity under the different types of starvation, as proposed previously (Zundel et al., 2009; Fessler et al., 2020).

## 2. Materials and methods

### 2.1. Strains, media, and growth conditions

The bacterial strains and plasmids used in this study are listed in Table 1. Except for MAS1074, all strains derive from wild-type strain *E. coli* K12 MAS1081(Stenum et al., 2017). The strain MAS1074 (BL21(DE3)-derived) carries pET11a(*selC*), which overproduces the rare tRNA^selC^ when induced with IPTG. MAS1074 was used as the spike-in strain for normalization of RNA quantification (Stenum et al., 2017). All cultures were grown in MOPS minimal medium (Neidhardt et al., 1974) supplemented with 0.2% glucose or 0.4% glycerol as specified. The auxotrophic strains MAS1190 and REP37 were additionally supplemented with 50 μg/ml arginine, 20 μg/ml leucine, and 10 μg/ml uracil. 100 μg/ml ampicillin was used when plasmid-bearing strains were cultured. Cells were grown in flasks at 37°C, shaking at 180 rpm. Growth was monitored by measurement of optical density at 436 nm (OD_436_) in a spectrophotometer. Cultures were grown for at least 10 generations to reach steady-state growth before sampling. Cells were phosphate-starved by filtration at 37°C using a 0.45 μm filter and washed twice with phosphate-free medium. The filter was resuspended in preheated, phosphate-free medium. When specified, 1 mM IPTG was added for induction of the *rpsA’-‘lacZ* allele carried on pIV18 (Vind et al., 1993), or for overexpression of tRNA^selC^ in MAS1074. For cell viability measurements, samples were serially diluted, plated on YT agar (0.8% tryptone, 0.5% yeast extract, 0.5% NaCl, 1.5% agar) plates with 100 μg/ml ampicillin, and incubated overnight at 37°C. Next day, visible colonies were counted and reported as colony forming units (CFU) per milliliter of undiluted culture.

**Table 1:** List of strains and plasmids used in this study.

Bacterial strains	Description	Source
MAS1074	BL21(DE3)-derived, carrying plasmid pET11a(*selC*)	Stenum et al. (2017)
MAS1081	MG1655 *rph^+^ gatC^+^ glpR^+^*	Gummesson et al. (2020)
MAS1143	MAS1081 *lacI*^q1^, *lacZ*::Tn*5*	This work
MAS1190	MAS1081 *ΔargG, ΔleuA, ΔpyrE::tet*	Prossliner et al. (2022)
REP37	MAS1081 *ΔargG, ΔleuA, ΔpyrE::tet, lacI^q1^, lacZ::Tn5*	This work
Plasmids		
pET11a	Expresses tRNA^selC^ from an IPTG-inducible promoter	Stenum et al. (2017)
pIV18	Expresses hybrid *rpsA’-‘lacZ* mRNA from an IPTG-inducible promoter	Vind et al. (1993)

### 2.2. Measurements of protein and nucleic acid accumulation by radioactive labeling

The two auxotrophic strains, MAS1190 and REP37 were grown in supplemented MOPS minimal medium as described above, additionally containing 0.18 μCi/ml ^3^H-leucine and 0.15 μCi/ml ^14^C-uracil. Samples were harvested every 30 min until the culture reached an approximate OD_436_ of 0.4–0.5. Cultures were then filtrated and resuspended in the same medium without phosphate. Samples were harvested every 10 min after phosphate was removed. Total protein (^3^H-leucine incorporated) and total nucleic acids (^14^C-uracil incorporated) were precipitated with 5% TCA at 0°C for at least 30 min. Samples for total DNA (also ^14^C-uracil incorporated) were treated with 0.5 M NaOH at 0°C for 16 h to hydrolyze RNA, followed by precipitation with 5% TCA at 0° C for at least 30 min. Precipitated TCA samples were filtrated onto glass fiber filters (Advantec) by vacuum filtration, washed three times with 5 ml ice-cold 5% TCA and dried overnight at room temperature (RT) before addition of 5 ml Ultima Gold™ scintillation cocktail (Perkin Elmer) and measuring counts per minute (cpm) by liquid scintillation counting.

Alternatively, protein accumulation was measured by ^35^S-methionine incorporation. The strain MAS1143/pIV18 was cultured to balanced growth. For volume normalization, 0.1 μCi/ml ^3^H-lysine was added and then chased after one generation with 10 μg/ml of non-radioactive lysine. At OD_436_ of 0.4–0.5, cells were P-starved by filtration and resuspension in the same medium without phosphate. Immediately after P starvation, the culture was split into two identical flasks containing 1 μCi/ml ^35^S-methionine and 75 μg/ml of non-radioactive methionine. One of the two cultures was then induced with 1 mM IPTG 9 min into starvation. Samples were harvested every 10 min, and radioisotope incorporation into total protein was measured by TCA-precipitation followed by determination of the ^35^S and ^3^H radioactivity of each sample in a scintillation counter.

### 2.3. Measurement of protein synthesis by pulse-chase analysis and 2D gel electrophoresis

Pulse-chase experiments for total protein labeling and separation by 2-dimensional (2D) gel electrophoresis were performed as previously described (Sørensen and Pedersen, 1998). The strain MAS1143/pIV18 was grown in balanced exponential growth, as described above. For volume normalization, 0.1 μCi/ml ^3^H-lysine was added and then chased after one generation with 10 μg/ml of non-radioactive lysine. At OD_436_ of 0.4–0.5, cells were phosphate-starved by filtration. Immediately after phosphate starvation, the culture was split into two identical flasks, and only one of them was induced with 1 mM IPTG at time 5 min. Samples for pulse-chase were taken by moving 2 ml of culture to a test tube, adding 10 μCi/ml of ^35^S-methionine for 30 s labeling and then chased with an excess (100 μg/ml) of non-radioactive methionine for 2 min. 0.5 ml of the pulse-chased culture were TCA-precipitated as described above to account for incorporation of radioactivity into total protein in each sample. The remainder of the culture was harvested into ice-cold tubes containing 50 μl of 50 mg/ml of chloramphenicol. After centrifugation, cell pellets were boiled in SDS buffer to obtain the total protein extracts. Proteins were separated by equilibrium 2D gel electrophoresis, as previously described (O’Farrell, 1975; Sørensen and Pedersen, 1998). Two percentages (7.5% and 10.5%) of polyacrylamide were used. Individual proteins (DnaK, ribosomal protein S1, GroEL) were identified by visualization in autoradiograms, and then extracted from the gel by protease digestion (Subtilisin). ^35^S and ^3^H counts of each protein at different time points were measured in a scintillation counter and divided by the ^35^S/^3^H ratio in TCA-precipitated total protein of each sample, to normalize for variations in ^35^S-methionine pulse sizes.

### 2.4. Spike-in, RNA extraction, and northern blot analysis

As described above, the strain MAS1081/pIV18 was grown in balanced exponential growth, and at OD_436_ of 0.4–0.5, cells were phosphate-starved by filtration. Shortly after phosphate starvation, the culture was split into two identical flasks, and only one of them was induced with 1 mM IPTG at the 5 min time point after filtration. Samples for RNA extraction were harvested in 1/6 volume of stop solution, consisting of 5% water-saturated phenol in ethanol, and kept at 0°C until all samples had been collected (Bernstein et al., 2002). A volume of spike-in culture equivalent to 5% of sampled cells was added to each sample from balanced growth based on the OD_436_ (Stenum et al., 2017). Since OD measurements do not correspond directly to cell numbers in starved cultures (Koch, 1970; Fessler et al., 2020), the phosphate-starved samples all received a fixed ratio of spike-in culture volume to sampled culture volume, which corresponded to 5% spike-in cells relative to cells in the sample harvested immediately upon filtration and resuspension in phosphate-free medium. For this reason, the relative levels of rRNA quantified by northern blot to changes in rRNA per volume of culture, irrespective of changes to the OD of the culture that occurred in the phosphate-free medium. RNA was extracted using a hot phenol protocol as previously described (Gummesson et al., 2020). RNA was separated on 1.5% agarose, 6% formaldehyde gels. Gels were blotted onto Hybond N^+^ (GE Healthcare) membranes by capillary transfer overnight. RNA was immobilized by UV irradiation at 1,200 μJ/cm^2^ and hybridized sequentially with ^32^P end-labeled DNA-oligo probes with the sequences: 23S 5′-GACCCATTATACAAATACGC, 16S 5′-AAGGAGGTGATCCAACCGCA, 5S 5′-ACACTACCATCGGCGCTAC, tRNA^selC^ 5′-ATTTGAAGTCCAGCCGCC. After washing, blots were exposed to phospho-storage screens and a Typhoon phosphorimager FLA7000 (GE Healthcare) was used to collect the radioactive signal. Quantification of the radioactivity of probed bands was done using ImageQuant TL 8.2. The quantified intensity of each band was divided by the intensity of the tRNA^selC^ band for spike-in normalization. This ratio was plotted relative to the average of the three reference samples harvested immediately before P starvation.

### 2.5. Reverse transcription quantitative PCR

Relative mRNA levels were measured by reverse transcription quantitative PCR (RT-qPCR) of the same RNA samples used for northern blots of ribosomal RNA. For this, RNA samples were treated with DNase I (Thermo Scientific) for 30 min at 37°C and phenol-extracted. Between 0.6 and 1.0 μg of RNA was used for reverse transcription using Thermo Scientific RevertAid RT Kit with the supplied random hexamer primers. To check for genomic DNA contamination, a reaction without the reverse transcriptase was included for every sample. 1/50 of the total cDNA of each sample was analyzed in triplicate by RT-qPCR using SsoAdvanced Universal SYBR Green Supermix (Bio-Rad) in the QuantStudio 3 system (Applied Biosystems). The thermal cycling program was 30 s at 95°C, followed by 40 cycles of 15 s at 95°C and 1 min at 60°C. For detection of *rpsA* cDNA only, the elongation temperature was instead 62.5°C. To check for amplification artifacts, a melting curve was performed with a final cycle of 15 s at 95°C, 1 min at 60°C, and a dissociation step to 95°C with 0.15°C/s increments. The primer sequences for target genes (*dnaK*, *groL*, *rpsA*, *lacZ*) and control gene (*selC*) were the following: dnaK-fw 5-CCACGCCTTCTATCATTGCC, dnaK-rv 5′-ACAGAGTGTTTTGCGGGTTC, groL-fw 5′-GCTATGCTGCAGGATATCGC, groL-rv 5′-CAACACGTTTAGCCTGACCC, lacZ fw 5′-TTTGCCGTCTGAATTTGACCTGAGCG, lacZ rv 5′-TCTTCCAGATAACTGCCGTCACTCCAG, rpsA fw 5′-TGACCAACCTGACCGACTAC, rpsA rv 5′-CAACTTTGGACGGGTGGATG, selC-fw 5′-GGCGGCTGGACTTCAAATC, selC-rv 5′-CGGAAGATCACAGGAGTCGA. The relative mRNA levels were calculated by normalizing to the control RNA (*selC*) expressed from the spike-in cells (MAS1074) following the formula E_target_^ΔCt^/E_control_^ΔCt^. These values were then normalized to the average of steady-state triplicate samples.

## 3. Results

### 3.1. Phosphate starvation was reflected in strongly reduced macromolecular synthesis rates and a net loss of RNA

**Figure 1:**
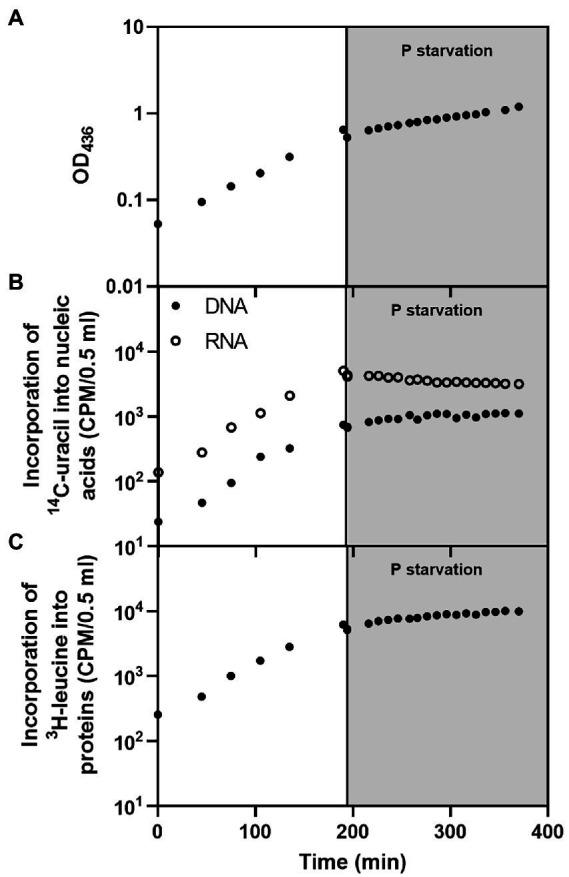
Macromolecular profile of *E. coli* MAS1190 (Δ*argG* Δ*leuA* Δ*pyrE::tet*) during balanced growth (white background) and after filtration into P-free medium (gray background). The panels show **(A)** optical density of the culture, **(B)** ^14^C-uracil incorporated into DNA (filled circles) and RNA (open circles), and **(C)** ^3^H-leucine incorporated into protein. The data set presented in this figure is representative of three independent biological replicates with similar results. The replicate experiments are shown in Supplementary Figure S1. Cells grew with a doubling time of ~57 min prior to starvation. The minor drop observed in all measurements immediately upon starvation was caused by the loss of cells during filtration. Note the logarithmic scale on the Y-axes.

To characterize the *E. coli* response to P starvation in terms of macromolecular synthesis, the overall replication, transcription, and translation activities before and after P starvation were monitored. We measured incorporation of radioactively labeled amino acid (^3^H-leucine) and nucleobase (^14^C-uracil) into proteins and nucleic acids, respectively, in a derivative of our *E. coli* wildtype strain (MAS1190 Δ*argG* Δ*leuA* Δ*pyrE::tet*) that is auxotrophic for leucine, arginine and uracil. The bacteria were cultured in MOPS minimal medium supplemented with glucose, uracil, leucine, and arginine as described in Methods. Prior to starvation, the cultures were in balanced growth, where protein, DNA and RNA accumulated at the same rate as the OD of the culture increased (Figure 1; Supplementary Figure S1). Steady-state growth was abruptly disrupted by rapid filtration of the cells into P-free medium. The effect of P starvation was most readily apparent in the RNA, which transitioned from exponentially growing RNA accumulation to a net loss of RNA from the culture in the hours after removal of P from the medium (Figure 1B). This finding agrees with the recently described fast degradation kinetics of rRNA, which constitutes the majority of cellular RNA, during short-term P starvation (Fessler et al., 2020).

The culture’s DNA contents continued to increase in the first hour after starvation before plateauing (Figure 1B), in line with the observation that stationary phase cells prioritize completion of replication to yield intact copies of chromosomes (Akerlund et al., 1995). Since the pools of free nucleotides are turned over within seconds in the cell (Chapman et al., 1971), the triphosphate nucleotides required to complete DNA replication must mainly arise from the breakdown of RNA, through the interconversion of ribonucleotides to deoxyribonucleotides (Bolton and Reynard, 1954; Bremer and Dennis, 2008; Jensen et al., 2008) in combination with further breakdown of ribonucleotides that releases inorganic phosphate for the generation of deoxyribonucleotide triphosphates (Rinas et al., 1995).

Protein synthesis continued after starvation, as seen by a net gain of protein in the culture for more than 2 h into the starvation period, albeit at a slower rate than before starvation (Figure 1C). The slight increase in accumulation of proteins correlates with the increase in OD (compare Figures 1A–C) and suggests that the OD increase during P starvation is caused mainly by continued protein synthesis. To our knowledge, neither a phenomenological nor a molecular theory has been developed to quantitatively predict how substantial the drop in protein synthesis rate would be upon removal of P. Moreover, it is not known which component of the translational apparatus first becomes limiting for protein synthesis under the P starvation condition.

### 3.2. Introduction of a long-lived mRNA resulted in increased protein synthesis during phosphate starvation

**Figure 2:**
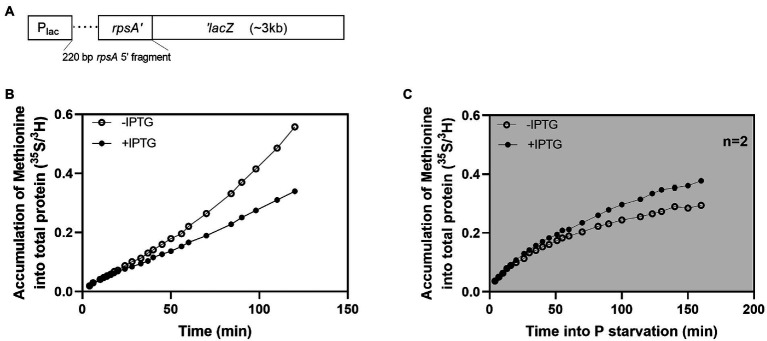
Induction of a long-lived hybrid mRNA led to an increased protein synthesis rate during P starvation. Effects of induction of a long-lived hybrid mRNA on protein synthesis under balanced growth and P starvation in *E. coli* strain MAS1143 (*lacI^q1^ lacZ*::Tn*5*). **(A)** Schematic showing the *rpsA’*-‘*lacZ* hybrid gene construct (not to scale). **(B)** Accumulation of ^35^S-methionine in protein during balanced growth (open circles) and after induction of the *rpsA’*-‘*lacZ* mRNA with IPTG (closed circles) in *E. coli* strain MAS1143/pIV18. The ratio of ^35^S/^3^H counts is presented over time. At time “0”, ^35^S-methionine was added to a culture in balanced growth that was pre-labeled with ^3^H-lysine. The culture was divided into two flasks, and IPTG was added to one of them at time +9 min. The data in panel B is representative of two independent biological replicates. The replicate experiment is shown in Supplementary Figure S2. **(C)** Accumulation of ^35^S-methionine in protein during P starvation, with (closed circles) or without (open circles) induction of *rpsA’-‘lacZ* transcription with IPTG in *E. coli* strain MAS1143/pIV18. Procedure as described for panel B, except that cells were transferred to P-free medium by filtration at time −2 min. The data in panel C corresponds to the average of two biological replicates, and the error bars (smaller than plotting symbols) indicate the SEM. The cultures used in panel B and C were grown in MOPS minimal medium supplemented with 0.4% glycerol and 100 μg/ml ampicillin. The average doubling time during balanced growth was 100 min.

To address the latter question of the limiting factor for protein synthesis, we asked if the protein synthesis rate would change if we made changes to the mRNA pool. We used a previously described hybrid mRNA, which is expressed as an *rpsA’*-‘*lacZ* translational fusion from plasmid pIV18(Vind et al., 1993; Figure 2A). In the hybrid mRNA, a 220 bp fragment, including the 5′ UTR and the 20 first codons of an *rpsA* expression-up mutant is inserted at the sixth codon of *lacZ*. This modification extends the functional half-life of the *lacZ* mRNA from 2 to 6 min. The expression of the hybrid mRNA is inducible by IPTG, and because the mRNA is highly expressed, has an extended half-life, a strong ribosomal binding site, and encodes a long open reading frame, it causes a redirection of ribosomes so that β-galactosidase produced from the mRNA takes over 25% of the total protein synthesis when the promoter is fully induced in unstarved cells (Vind et al., 1993).

We measured protein production in strain MAS1143 (*lacI*^q1^ *lacZ*::Tn*5*) carrying pIV18 during balanced growth and after P starvation. A culture of MAS1143/pIV18 in balanced growth was first labeled with ^3^H-lysine for one generation for volume normalization. The culture was split in two and *rpsA’-‘lacZ* expression was induced in one of them by addition of IPTG. Protein accumulation was measured using ^35^S-methionine, which was added with excess methionine when the cultures were split. When the expression of the long-lived mRNA was induced in exponentially growing cells, protein production was initially undisturbed, but within 30 min, the rate of incorporation of ^35^S-methionine into protein became slower than that of the uninduced culture (Figure 2B; Supplementary Figure S2). The production of unnecessary protein, such as β-galactosidase, has been reported to reduce growth rate due to the inability of such cells to allocate sufficient resources to production of ribosomes and other translation machinery to sustain rapid growth (Scott and Hwa, 2011; Basan et al., 2015). Similarly, by claiming a large fraction of the translating ribosomes, the *rpsA’-‘lacZ* mRNA reduces translation of other mRNAs, including those encoding proteins of the translational apparatus, leading to an overall reduction in the protein synthesis capacity of the cells (Vind et al., 1993).

Intriguingly, the induction of the *rpsA’-‘lacZ* mRNA had a radically different effect on protein synthesis in P-starved cells. When transcription of the hybrid mRNA was induced after P starvation, we observed an increase in protein production compared to the uninduced culture (Figure 2C). Specifically, protein accumulation slowed down upon P starvation in both cultures, but the culture expressing *rpsA’-‘lacZ* maintained a higher level of protein synthesis than the uninduced culture for at least 2 h.

**Figure 3:**
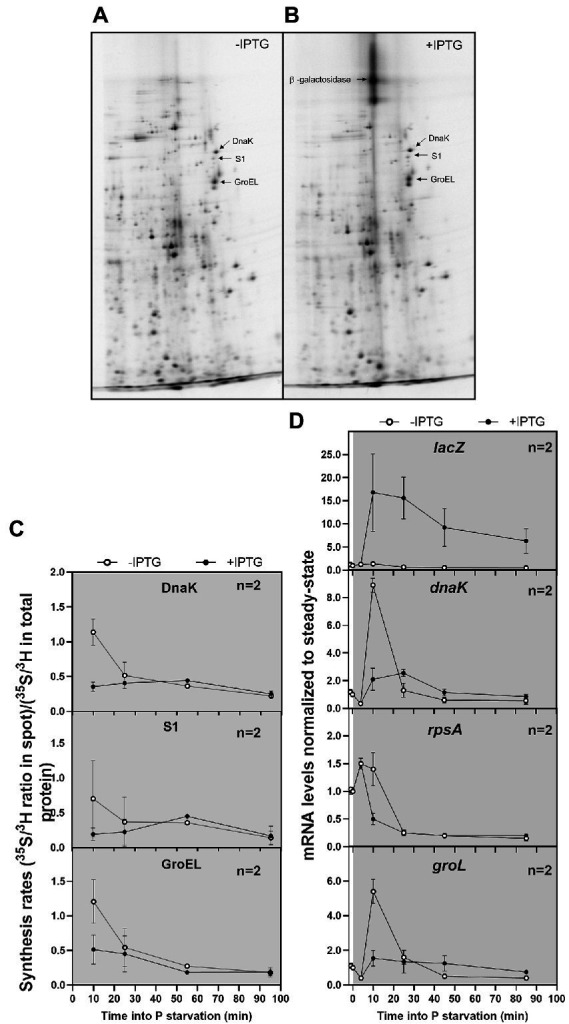
Effects of hybrid mRNA induction on expression of endogenous mRNA and protein. **(A)** Expression pattern of *E. coli* MAS1143/pIV18 proteins from pulse labeling after 55 min of P starvation, shown on a 10.5% 2D PAGE gel. **(B)** As panel A, except IPTG was added to the culture at time + 5 min. **(C)** Expression levels of ribosomal protein S1, GroEL and DnaK determined from 2D-gels of pulse labeling experiments made at the times indicated, like the examples shown in panel A and B. Autoradiograms from additional time points are shown in Supplementary Figure S3. The ratio of ^35^S/^3^H shows the relative synthesis rate during P starvation, with (closed symbols) or without (open symbols) induction of the *rpsA’*-‘*lacZ* hybrid mRNA. Each sample was run on an independent gel for protein separation and identification. **(D)** Relative mRNA levels quantified by RT-qPCR of total RNA harvested from MAS1143/pIV18 before and after P starvation. After starvation, the culture was split, and IPTG was added to one of the cultures at *t* = 5 min. Five per cent spike-in cells overexpressing the rare tRNA^selC^ were added for normalization (see Methods). The values shown in panels C-D correspond to the average of two biological replicates, and the error bars indicate the SEM. The cultures used in panels A-D were grown in MOPS minimal medium supplemented with 0.4% glycerol and 100 μg/ml ampicillin. The average doubling time during balanced growth was 100 min.

**Figure 4:**
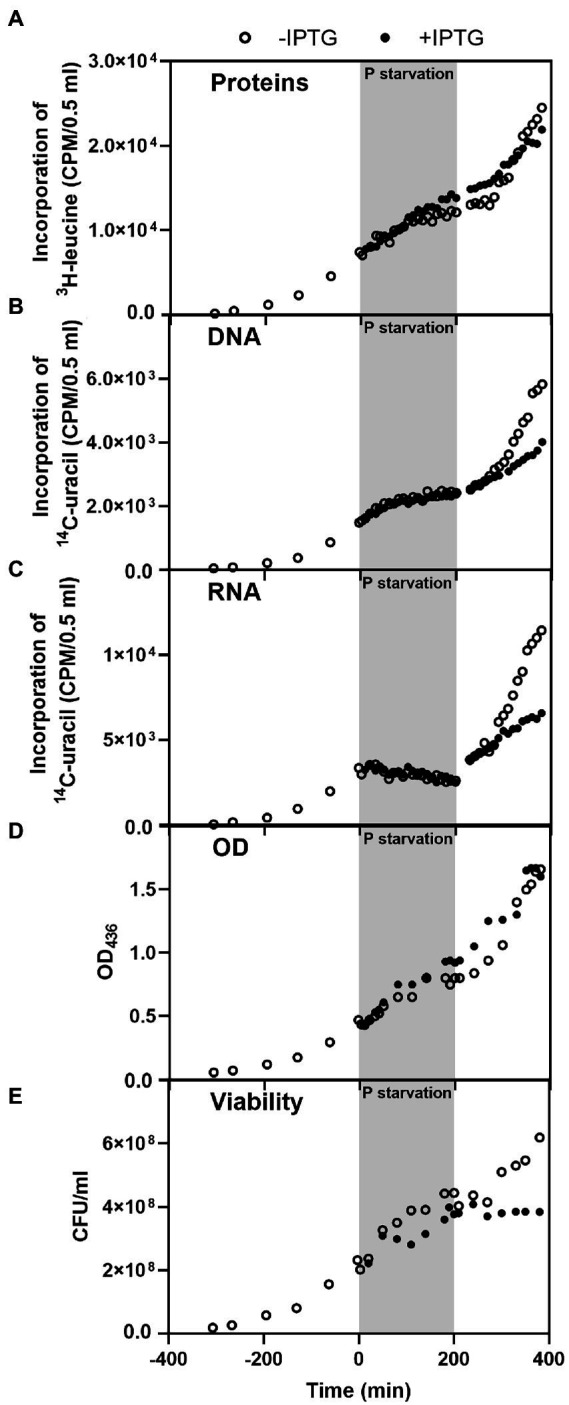
Production of unnecessary protein decreased *E. coli*’s ability to recover from P starvation. Macromolecular profile of *E. coli* strain REP37/pIV18 (*ΔargG ΔleuA ΔpyrE::tet lacI^q1^ lacZ::Tn5*) with or without induction of long-lived hybrid mRNA during P starvation (gray background) and refeed (white background). The panels show accumulation of **(A)** protein, **(B)** DNA, **(C)** RNA, panel **(D)** shows optical density, and **(E)** cell viability as CFU/ml. The data shown in this figure is representative of two independent biological replicates. The replicate experiment is shown in Supplementary Figure S4. The cultures were grown in MOPS minimal medium supplemented with 50 μg/ml arginine, 20 μg/ml leucine, 10 μg/ml uracil, 0.4% glycerol and 100 μg/ml ampicillin. The average doubling time in steady state was 100 min. Note the linear scale on the Y-axes.

To investigate whether the synthesis of β-galactosidase occurred at the expense of the synthesis of other proteins, we performed pulse-chase labeling of newly synthesized proteins followed by two-dimensional gel electrophoresis. As in the previous labeling experiment, a culture of MAS1143/pIV18 in balanced growth was first labeled with ^3^H-lysine for volume normalization. Immediately after P starvation, the culture was split in two, and expression of *rpsA’-‘lacZ* was induced with IPTG in one of them. Aliquots of the cultures were used for pulse-chase labeling with ^35^S-methionine at different time points after the addition of IPTG and the proteins were separated on two dimensional-polyacrylamide gels as shown for the time point 55 min into P starvation in Figure 3A (uninduced) and Figure 3B (induced). Additional autoradiograms from time point 10, 25, and 95 min are shown in Supplementary Figure S3. We focused our analysis on the first hours of P starvation, and did not include autoradiograms of steady-state samples, since P starvation is known to induce substantial changes to the proteome, independent of hybrid mRNA availability (Groat et al., 1986; Vanbogelen et al., 1996). Induction of the hybrid mRNA resulted in production of more β-galactosidase than any other protein that could be detected on the gels but did not appear to gravely disturb the expression levels of the majority of other proteins since neither the intensities of the individual spots nor the spot pattern changed substantially (compare panel A and B in Supplementary Figure S3). We also did not observe any indication of mistranslation, which can often be detected as “stuttering spots” on 2D-gels (Parker et al., 1978). In particular, the expression of three specific proteins (ribosomal protein S1, chaperone DnaK and chaperonin GroEL) was previously shown to suffer from ribosome deficiency upon induction of the hybrid *lacZ* mRNA during exponential growth (Vind et al., 1993). The incorporation of ^35^S-methionine into these three endogenous proteins under P starvation was determined by cutting the protein spots out of the gels, followed by digestion of the protein and determination of the isotope ratio by liquid scintillation counting. The relative synthesis rates ((^35^S/^3^H in spot)/(^35^S/^3^H in total protein)) of the selected proteins in the induced and uninduced samples are shown in Figure 3C. At the first time point, 10 min after starvation, we observed a reduction in synthesis of DnaK, GroEL, and S1 in the induced culture relative to the uninduced counterpart, as was previously observed under unstarved conditions (Vind et al., 1993). However, there were no substantial differences in the relative synthesis rates of these three proteins for the remainder of the starvation period. Thus, expression of β-galactosidase from the long-lived *rpsA’-‘lacZ* transcript occurred at the expense of endogenous proteins during early starvation, but once *E. coli* had adapted to P starvation, the exogenous *rpsA*’-‘*lacZ* gene was expressed in addition to the endogenous genes (Rinas et al., 1995), suggesting that auxiliary capacity for protein expression was available in the starvation-adapted cells. This interpretation of the data was validated at the mRNA level by RT-qPCR. As can be seen in Figure 3D, induction of *rpsA’-‘lacZ* with IPTG 5 min into P starvation resulted in >15-fold up-regulation of the hybrid mRNA, and was accompanied by a brief relative reduction in *dnaK* and *groL* mRNAs shortly after P starvation, but ~25 min after P removal the mRNA levels of *dnaK*, *groL* and *rpsA* in the induced culture closely resembled those of the uninduced culture. The similar production rates of DnaK, S1 and GroEL protein (Figure 4C) from similar quantities of mRNA (Figure 3D) in starvation-adapted cells shows that translation initiation on these mRNAs is not affected by the presence of high levels of the *rpsA’-‘lacZ* transcript. Together, these measurements show that protein synthesis upon P starvation does not operate at an absolute capacity maximum determined by, e.g., the total P available in the culture, but is amenable to increase if additional mRNA is available for translation.

Next, we assessed the macromolecular synthesis profile of a derivative of our wildtype *E. coli* strain REP37 (Δ*argG* Δ*leuA* Δ*pyrE*::*tet lacI^q1^ lacZ*::Tn*5*) with the hybrid mRNA plasmid (REP37/pIV18). As in Figure 1, we measured accumulation of protein, DNA and RNA of REP37/pIV18 (Figure 4). While total protein synthesis clearly increased during phosphate starvation upon induction of the hybrid mRNA (Figures 2, 4A), we could not detect a difference in the DNA accumulation between the induced or uninduced cultures (Figure 4B), indicating that the β-galactosidase-producing cells complete DNA replication at the expense of total RNA under P starvation, like wildtype cells (Figure 1B; note that Figures 1, 4 are shown on different scales). The rates of loss of ^14^C-uracil-labeled RNA also appeared very similar with and without induction of the hybrid mRNA (Figure 4C). We return to this point in the Discussion.

As could be expected from the behavior of cultures in balanced growth upon induction of the hybrid mRNA (Vind et al., 1993; Figure 2B), the induced cultures did not recover as well as the uninduced cultures when P was supplied after a period of starvation. Figures 4A–C show reduced rates of macromolecular synthesis in the induced cultures compared to their uninduced counterparts after P refeed. In fact, despite some macromolecular synthesis and thus increased optical density upon refeed with P (Figure 4D), the number of viable cells in the induced cultures did not increase for several hours after P refeed (Figure 4E).

Our results show that the availability of the hybrid mRNA during P starvation enables *E. coli* to produce protein at an increased rate. The results suggest that the limiting factor for protein production during P starvation in wildtype cells is not directly the lack of ribosomes, ternary complexes or GTP, but that protein synthesis is limited by regulatory events and substrate availability that lead to reduced amounts of mRNA.

### 3.3. rRNA degradation in cells expressing unnecessary protein

**Figure 5:**
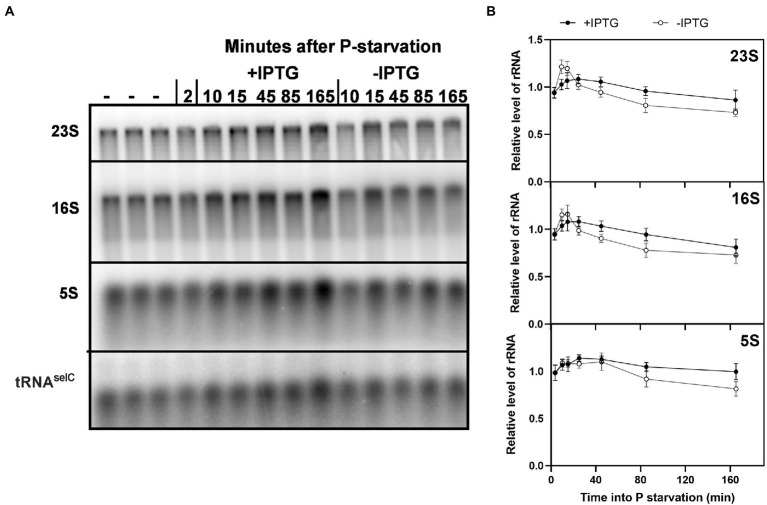
Induction of a long-lived hybrid mRNA resulted in higher levels of full-length rRNA during P starvation. **(A)** A 1.5% agarose denaturing gel was used for separation of RNA samples from *E. coli* strain MAS1143 (*lacI*^q1^ *lacZ*::Tn5)/pIV18 before (–) and after P starvation. After starvation, the culture was split, and IPTG was added to one of the cultures at *t* = 5 min. Five per cent spike-in cells overexpressing the rare tRNA^selC^ were added to each sample for normalization (see Methods). The RNA was transferred from the agarose gel to a Hybond N+ membrane, and probed for 23S, 16S, 5S, and tRNA^selC^. **(B)** Quantification of full length 5S, 16S, and 23S normalized to tRNA^selC^ are shown relative to the average of the three RNA samples harvested during balanced growth prior to starvation. The shown values correspond to the mean of five independent biological replicates, and the error bars indicate the SEM.

P starvation leads to extensive degradation of rRNA (Fessler et al., 2020; Himeoka et al., 2022; Prossliner et al., 2022). We wondered if the increased protein synthesis activity in the presence of the hybrid mRNA might affect the rate of rRNA degradation. We therefore used northern blots to measure the relative levels of full length 23S, 16S, and 5S rRNA under P starvation in the presence or absence of the long-lived *rpsA’-‘lacZ* transcript. Figure 5 shows that rRNA was degraded both in the induced and uninduced samples, which explains the overall loss of RNA shown in Figures 1, 4C. However, rRNA levels remained higher in the cells that are expressing the hybrid mRNA. The observed differences (13% for 23S and 16S rRNA, and 18% for 5S rRNA after 2 h of starvation) were minor but consistent throughout all biological replicates (*n* = 5). The results suggest that rRNA degradation under P starvation is controlled at least in part by the degree to which the ribosomes are engaged in translation, as proposed also for carbon starvation (Zundel et al., 2009).

## 4. Discussion

Based on a hypothesis of optimal resource allocation, *E. coli* is expected to redistribute cellular P upon depletion of extracellular sources to the extent that it would maximize evolutionary fitness. The optimal outcome is not well defined, and there are several possibilities, such as maximizing survival time during P starvation, minimizing lag time upon P refeed or obtaining a maximal protein synthesis per unit P during starvation. The optimal outcome is likely some combination of these possibilities, depending on the specific circumstances of the P limitation. Redistribution of P would occur between RNA and the other P-rich compounds such as DNA and cell membrane, as well as within the RNA fraction to optimize the size of the sub-fractions rRNA, tRNA and mRNA. Several studies suggest that the total mass of the translation apparatus, including the relative size of the rRNA and tRNA fractions, is indeed subject to careful tuning by evolution. For example, experiments with hyper- and hypo-accurate ribosomes have shown that the processivity of the ribosomes, at the cost of accuracy, is an important parameter to save investment in ribosome mass (Kurland and Ehrenberg, 1984) as is the bias in codon usage in highly expressed genes (Ehrenberg and Kurland, 1984). Strikingly, it was recently demonstrated that there is an accurate correspondence between the experimentally measured quantities and the computed theoretical minimum for total dry mass allocated to the protein synthesis machinery that could achieve a given protein synthesis rate (Hu et al., 2020). With respect to the relative size of the rRNA and tRNA pools specifically, it has been observed repeatedly that the ratio of ternary complexes (charged tRNAs bound to EF-Tu⋅GTP) to ribosomes increases at decreasing steady-state growth rates [see Scott et al., 2014 and references therein]. The growth-rate-dependence of this ratio was faithfully replicated theoretically by computing the minimal sum of ribosome and ternary complex masses (RNA and protein) that would yield the protein production rates characteristic for each growth rate (Hu and Lercher, 2021). Furthermore, the optimal ratio of ternary complexes to ribosomes at different steady-state growth rates was proposed to be hard-wired into the genome of fast-growing bacteria, arising as a direct consequence of the relative location of rRNA and tRNA genes relative to the origin of replication (Hu and Lercher, 2021).

If similar adaptive changes to the relative sizes of the RNA pools occur dynamically upon nutrient starvation, it would need to occur at the level of differential RNA degradation rates, since rRNA and tRNA synthesis is strongly reduced upon starvation (reviewed in Dennis et al., 2004). Indeed, tRNA is exceedingly stable under C, N, and P starvation (Prossliner et al., 2022), while starvation triggers rRNA breakdown to different extents depending on the type of nutrient starved for (Deutscher, 2003; Fessler et al., 2020; Prossliner et al., 2022).

The extensive degradation of rRNA during P starvation offers a fitness advantage by releasing nucleotide mono- and diphosphates that are salvaged to regenerate nucleotide triphosphates in the absence of an external P source. An extreme example of how degradation of ribosomes can allow growth under P starvation was seen for *E. coli* grown in a P-free medium containing arsenate (Basturea et al., 2012). Arsenate triggered massive ribosome break down, which served as a P source that allowed a small subset of arsenate-resistant cells in the culture to continue growth, albeit very slowly. An intriguing question is how cells regulate the extent of rRNA breakdown that is appropriate for a given starvation condition.

Here, we studied the extreme condition of abrupt removal of extracellular P by filtration of *E. coli* cultures in balanced growth into P-free medium. We observed a net loss of RNA from the P-starved cells, which was mainly caused by degradation of rRNA (Figure 1B, 5; Fessler et al., 2020). The RNA loss was concomitant with a net gain of DNA, illustrating dynamic resource allocation. Protein synthesis continued upon P depletion at a reduced rate relative to steady-state growth (Figure 1C). To study which factor(s) limits the starved cultures’ protein synthesis rate, we made use of a hybrid *rpsA’-‘lacZ* mRNA, which supports abundant translation of β-galactosidase in cultures of *E. coli* that are nutrient replete. The hybrid mRNA has a long lifetime, a strong ribosome binding site, a long open reading frame, and translation is not feedback inhibited by S1 protein as it is for *rpsA* mRNA (Vind et al., 1993). Thus, one molecule of the hybrid mRNA can support many more amino-acid-incorporation events than an average endogenous mRNA molecule. A key finding from this study is that induction of the hybrid mRNA under P starvation led to an increased protein synthesis rate. Furthermore, the synthesis of individual proteins was largely unaffected by the induction of the hybrid mRNA once the cells had adapted to the P starvation condition (Figures 3A–C; Supplementary Figure S3). These observations suggest that once the cells have adapted to P starvation, they are operating below their capacity for protein synthesis, limited by the availability of the mRNA template, rather than by lack of the catalyst (the ribosome) or the substrate for the reaction (the ternary complexes). Furthermore, the mRNA measurements (Figure 3D) indicate that the induction of the hybrid mRNA 5 min into phosphate starvation further reduced the availability of substrate to the RNA polymerase to a point where the stress response genes *dnaK* and *groL* had reduced mRNA synthesis at 10 min into the starvation, compared to the synthesis in the uninduced culture. At later time points there was no detectable difference between the individual mRNA pools and they appear to have reached a new stable level, except for the induced *rpsA’-‘lacZ* hybrid mRNA with the extraordinary long half-life (Figure 3D). We expect that the emergence of auxiliary capacity for protein synthesis coincides with degradation of the majority of the pre-starvation bulk mRNA.

Our results also support the model that rRNA breakdown upon starvation is tied to reduced protein synthesis, as proposed by the Deutscher group (Zundel et al., 2009). During C starvation, the initial rRNA cleavage occurs at the interface between the small and large ribosomal subunit, suggesting that rRNA breakdown occurs from free ribosomal subunits rather than assembled ribosomes (Zundel et al., 2009; Basturea et al., 2011; Sulthana et al., 2016). This finding led to the simple model that increased rRNA breakdown may be a direct consequence of reduced protein synthesis activity under starvation, which leads to an increase in unengaged ribosomal subunits (Zundel et al., 2009). We observed higher rRNA levels in the P-starved cultures that synthesized more protein due to IPTG-induced expression of the hybrid mRNA, than in similar cultures without IPTG (Figure 5). The increase in rRNA levels was modest, ~10%–15%, and while the difference was reproducibly detected by northern blot probing of full length 5S, 16S, and 23S rRNA, we could not detect it in the ^14^C-uracil experiment shown in Figure 4C. A possible explanation for this discrepancy is that the total RNA content was indirectly calculated in the latter approach by measuring ^14^C-labeled total nucleic acid precipitation with TCA and subtracting the alkali-stable counts obtained from ^14^C-labeled DNA precipitated after sodium hydroxide treatment. This is a less targeted method than northern blotting, and does not discriminate between full-length and partially degraded RNA molecules, which may explain our inability to detect a difference between induced and uninduced cultures by ^14^C labeling.

**Figure 6:**
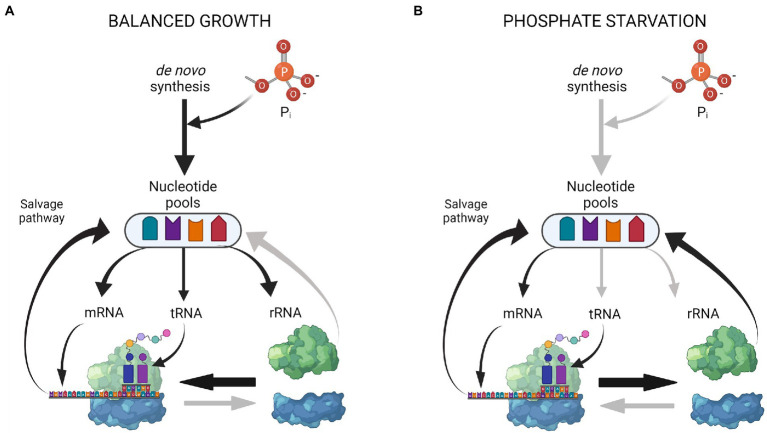
Coarse model of RNA pool redistribution upon P starvation. **(A)** During steady-state growth, the nucleotide pools are maintained by *de novo* synthesis and nucleotide recycling from mRNA turnover. Most ribosomes are engaged in translation. **(B)** During starvation, no extracellular P is available for *de novo* synthesis. RNA synthesis is decreased and focused on mRNAs of the Pho regulon. Reduced protein synthesis leads to more vacant ribosomes, which makes the RNA in the free ribosomal subunits susceptible to degradation. Nucleotides for continued mRNA synthesis are provided from rRNA degradation and mRNA turnover. The figure was created with Biorender.com.

Figure 6 summarizes the emerging picture of how resources within the RNA pool are redistributed upon P starvation. During balanced growth in minimal medium (Figure 6A), nucleotides are synthesized *de novo*, and a large flux is provided by recycling of the short-lived mRNA. Most ribosomes are engaged in translation and rRNA is therefore not subject to degradation. Upon P starvation (Figure 6B), phosphate for nucleotides can no longer be assimilated *de novo*, and overall RNA synthesis is strongly reduced, mainly by the action of (p)ppGpp on RNA polymerase and the reduced nucleotide triphosphate pools (Nazar et al., 1972; Spira et al., 1995). The reduction in mRNA availability results in an increase of free ribosomal subunits, making more rRNA subject to degradation. The nucleotides released from rRNA breakdown are used to sustain a reduced level of mRNA synthesis and protein synthesis during starvation. This simple model implies a dynamic rRNA degradation process, which is continuously adjusted by the degree to which ribosomes are engaged in protein synthesis. The levels of full-length rRNA eventually stabilize at a low level after ~8 h of P starvation (Himeoka et al., 2022; Prossliner et al., 2022). This may indicate the ‘tipping point’, at which additional mRNA would not lead to surplus protein synthesis, because ribosome availability becomes the limiting factor for protein synthesis, as it is during balanced growth (Vind et al., 1993). More experiments are needed to clarify if such a tipping point occurs during prolonged P starvation.

This present set of data contributes to a coarse yet simple hypothesis for the regulation of ribosome degradation during various stresses: ribosome content in different starvation conditions might be causally correlated with the availability of mRNA for translation. It has been known for decades that mRNA is limiting for translation during (early) amino acid starvation (Sørensen et al., 1994), similar to the observation here for P starvation. In the case of amino acid starvation, the naïve expectation would be that the protein synthesis rate is directly limited by the concentration of charged tRNA carrying the amino acid starved for. However, inhibition of the RNA polymerase by the elevated level of (p)ppGpp (Ross et al., 2013, 2016) produced by RelA at the starving ribosomes (Winther et al., 2018) reduces the mRNA pool considerably within minutes (Gummesson et al., 2020) and ribosomes become vacant due to lack of mRNA. Likewise, increased ppGpp production during early P starvation combined with the effect of substrate depletion in the form of low ribonucleotide triphosphate pools (Spira et al., 1995; Germain et al., 2019) reduces the RNA polymerase activity, resulting in low mRNA production, leaving a fraction of ribosomes idle. This interpretation agrees with the general thinking that bacterial stress responses are tuned to ensure that the available resources for protein synthesis under stress are concentrated on the successful production of a limited number of proteins, rather than permitting more ribosomes to initiate translation than what the available resources can support. For example, a *relA*^−^ mutant fails to limit mRNA availability upon amino acid starvation, and as a consequence of the increased protein synthesis activity, it suffers 10–40 fold greater reductions in tRNA charging, leading to increased stalling of ribosomes, mistranslation, and the inability to recover from starvation (Sørensen et al., 1994). Another example is that depletion of Mg^2+^ leads to extensive ribosome breakdown (McCarthy, 1962) and a halt in rRNA synthesis due to depletion of ATP and increased (p)ppGpp levels. Consequently, the available Mg^2+^ ions (~170 Mg^2+^ ions per ribosome) are concentrated on fewer, but functional, ribosomes. An interesting prediction from this simple view on the regulation of ribosome levels during starvation is that the total mRNA levels of cells cultured under different nutrient limitations might generally correlate with the observed ribosome levels (Li et al., 2018; Fessler et al., 2020).

## Data availability statement

The original contributions presented in the study are included in the article/Supplementary material, further inquiries can be directed to the corresponding authors.

## Author contributions

MS, RE, and SS designed experiments and interpreted the data. RE and MS performed the experiments. RE wrote the initial manuscript draft. MS and SS co-wrote and edited the manuscript. All authors contributed to the article and approved the submitted version.

## Funding

This work was supported by the Independent Research Fund Denmark, grants 8021-00280A and 8049-00071B.

## Conflict of interest

The authors declare that the research was conducted in the absence of any commercial or financial relationships that could be construed as a potential conflict of interest.

## Publisher’s note

All claims expressed in this article are solely those of the authors and do not necessarily represent those of their affiliated organizations, or those of the publisher, the editors and the reviewers. Any product that may be evaluated in this article, or claim that may be made by its manufacturer, is not guaranteed or endorsed by the publisher.

## References

[ref1] Akerlund T. Nordstrom K. Bernander R. (1995). Analysis of cell size and DNA content in exponentially growing and stationary-phase batch cultures of *Escherichia coli*. J. Bacteriol. 177, 6791–6797. doi: 10.1128/jb.177.23.6791-6797.1995, PMID: 7592469 PMC177544

[ref2] Basan M. Zhu M. Dai X. Warren M. Sévin D. Wang Y. P.. (2015). Inflating bacterial cells by increased protein synthesis. Mol. Syst. Biol. 11:836. doi: 10.15252/msb.20156178, PMID: 26519362 PMC4631207

[ref3] Basturea G. N. Harris T. K. Deutscher M. P. (2012). Growth of a bacterium that apparently uses arsenic instead of phosphorus is a consequence of massive ribosome breakdown. J. Biol. Chem. 287, 28816–28819. doi: 10.1074/jbc.C112.394403, PMID: 22798070 PMC3436571

[ref4] Basturea G. N. Zundel M. A. Deutscher M. P. (2011). Degradation of ribosomal RNA during starvation: comparison to quality control during steady-state growth and a role for RNase PH. RNA 17, 338–345. doi: 10.1261/rna.2448911, PMID: 21135037 PMC3022282

[ref5] Bernstein J. A. Khodursky A. B. Lin P. H. Lin-Chao S. Cohen S. N. (2002). Global analysis of mRNA decay and abundance in *Escherichia coli* at single-gene resolution using two-color fluorescent DNA microarrays. Proc. Natl. Acad. Sci. 99, 9697–9702. doi: 10.1073/pnas.112318199, PMID: 12119387 PMC124983

[ref6] Blanco A. G. Sola M. Gomis-Rüth F. X. Coll M. (2002). Tandem DNA recognition by PhoB, a two-component signal transduction transcriptional activator. Structure 10, 701–713. doi: 10.1016/S0969-2126(02)00761-X, PMID: 12015152

[ref7] Bolton E. T. Reynard A. M. (1954). Utilization of purine and pyrimidine compounds in nucleic acid synthesis by *Escherichia coli*. Biochim. Biophys. Acta 13, 381–385. doi: 10.1016/0006-3002(54)90345-5, PMID: 13140351

[ref8] Bremer H. Dennis P. P. (2008). Modulation of chemical composition and other parameters of the cell at different exponential growth rates. EcoSal Plus 3, 1–48. doi: 10.1128/ecosal.5.2.3, PMID: 26443740

[ref9] Cashel M. Gallant J. (1969). Two compounds implicated in the function of the RC gene of *Escherichia coli*. Nature 221, 838–841. doi: 10.1038/221838a0, PMID: 4885263

[ref10] Chapman A. G. Fall L. Atkinson D. E. (1971). Adenylate energy charge in *Escherichia coli* during growth and starvation. J. Bacteriol. 108, 1072–1086. doi: 10.1128/jb.108.3.1072-1086.1971, PMID: 4333317 PMC247190

[ref11] Davis B. D. Luger S. M. Tai P. G. (1986). Role of ribosome degradation in the death of starved *Escherichia coli* cells. J. Bacteriol. 166, 439–445. doi: 10.1128/jb.166.2.439-445.1986, PMID: 2422153 PMC214624

[ref12] Dennis P. P. Ehrenberg M. Bremer H. (2004). Control of rRNA synthesis in *Escherichia coli*: a systems biology approach. Microbiol. Mol. Biol. Rev. 68, 639–668. doi: 10.1128/MMBR.68.4.639-668.2004, PMID: 15590778 PMC539008

[ref13] Deutscher M. P. (2003). Degradation of stable RNA in bacteria. J. Biol. Chem. 278, 45041–45044. doi: 10.1074/jbc.R300031200 12941949

[ref14] Ehrenberg M. Kurland C. G. (1984). Costs of accuracy determined by a maximal growth rate constraint. Q. Rev. Biophys. 17, 45–82. doi: 10.1017/S0033583500005254, PMID: 6484121

[ref15] Fessler M. Gummesson B. Charbon G. Svenningsen S. L. Sørensen M. A. (2020). Short-term kinetics of rRNA degradation in *Escherichia coli* upon starvation for carbon, amino acid or phosphate. Mol. Microbiol. 113, 951–963. doi: 10.1111/mmi.14462 31960524

[ref16] Gardner S. G. McCleary W. R. (2019). Control of the phoBR regulon in *Escherichia coli*. EcoSal Plus 8, 1–20. doi: 10.1128/ecosalplus.ESP-0006-2019 PMC11573284 31520469

[ref17] Germain E. Guiraud P. Byrne D. Douzi B. Djendli M. Maisonneuve E. (2019). YtfK activates the stringent response by triggering the alarmone synthetase SpoT in *Escherichia coli*. Nat. Commun. 10:5763. doi: 10.1038/s41467-019-13764-4, PMID: 31848343 PMC6917717

[ref18] Groat R. G. Schultz J. E. Zychlinsky E. Bockman A. Matin A. (1986). Starvation proteins in *Escherichia coli*: kinetics of synthesis and role in starvation survival. J. Bacteriol. 168, 486–493. doi: 10.1128/jb.168.2.486-493.1986, PMID: 3536847 PMC213508

[ref19] Gummesson B. Shah S. A. Borum A. S. Fessler M. Mitarai N. Sørensen M. A.. (2020). Valine-induced isoleucine starvation in *Escherichia coli* K-12 studied by spike-in normalized RNA sequencing. Front. Genet. 11:144, 1–17. doi: 10.3389/fgene.2020.00144, PMID: 32211022 PMC7066862

[ref20] Himeoka Y. Gummesson B. Sørensen M. A. Svenningsen S. L. Mitarai N. (2022). Distinct survival, growth lag, and rRNA degradation kinetics during long-term starvation for carbon or phosphate. mSphere. 7, 1–12. doi: 10.1128/MSPHERE.01006-21 PMC9241543 35440180

[ref21] Hu X. P. Dourado H. Schubert P. Lercher M. J. (2020). The protein translation machinery is expressed for maximal efficiency in *Escherichia coli*. Nat. Commun. 11:5260. doi: 10.1038/s41467-020-18948-x, PMID: 33067428 PMC7568582

[ref22] Hu X. P. Lercher M. J. (2021). An optimal growth law for RNA composition and its partial implementation through ribosomal and tRNA gene locations in bacterial genomes. PLoS Genet. 17:e1009939. doi: 10.1371/journal.pgen.1009939, PMID: 34843465 PMC8659690

[ref23] Jensen K. F. Dandanell G. Hove-Jensen B. Willemoës M. (2008). Nucleotides, nucleosides, and nucleobases. EcoSal Plus 3, 1–68. doi: 10.1128/ecosalplus.3.6.2 26443734

[ref24] Klumpp S. Scott M. Pedersen S. Hwa T. (2013). Molecular crowding limits translation and cell growth. Proc. Natl. Acad. Sci. U. S. A. 110, 16754–16759. doi: 10.1073/pnas.1310377110, PMID: 24082144 PMC3801028

[ref25] Koch A. L. (1970). Turbidity measurements of bacterial cultures in some available commercial instruments. Anal. Biochem. 38, 252–259. doi: 10.1016/0003-2697(70)90174-0, PMID: 4920662

[ref26] Kurland C. G. Ehrenberg M. (1984). Optimization of translation accuracy. Prog. Nucleic Acid Res. Mol. Biol. 31, 191–219. doi: 10.1016/S0079-6603(08)60378-5 6397771

[ref27] Li S. H. J. Li Z. Park J. O. King C. G. Rabinowitz J. D. Wingreen N. S.. (2018). *Escherichia coli* translation strategies differ across carbon, nitrogen and phosphorus limitation conditions. Nat. Microbiol. 3, 939–947. doi: 10.1038/s41564-018-0199-2, PMID: 30038306 PMC6278830

[ref28] McCarthy B. J. (1962). The effects of magnesium starvation on the ribosome content of *Escherichia coli*. Biochim. Biophys. Acta, Spec. Sect. Nucleic Acids Relat. Subj. 55, 880–889. doi: 10.1016/0926-6550(62)90345-6

[ref29] Nazar R. N. Tyfield L. A. Wong J. T.-F. (1972). Regulation of ribonucleic acid accumulation in vivo by nucleoside triphosphates. J. Biol. Chem. 247, 804–798. doi: 10.1016/S0021-9258(19)45678-0 4550761

[ref30] Neidhardt F. C. Bloch P. L. Smith D. F. (1974). Culture medium for enterobacteria. J. Bacteriol. 119, 736–747. doi: 10.1128/jb.119.3.736-747.1974, PMID: 4604283 PMC245675

[ref31] O’Farrell P. H. (1975). High resolution two-dimensional electrophoresis of proteins. J. Biol. Chem. 250, 4007–4021. doi: 10.1016/S0021-9258(19)41496-8 236308 PMC2874754

[ref32] Parker J. Pollard J. W. Friesen J. D. Stanners C. P. (1978). Stuttering: high-level mistranslation in animal and bacterial cells. Proc. Natl. Acad. Sci. U. S. A. 75, 1091–1095. doi: 10.1073/pnas.75.3.1091, PMID: 349556 PMC411414

[ref33] Piir K. Paier A. Liiv A. Tenson T. Maiväli Ü. (2011). Ribosome degradation in growing bacteria. EMBO Rep. 12, 458–462. doi: 10.1038/embor.2011.47, PMID: 21460796 PMC3090016

[ref34] Prossliner T. Agrawal S. Heidemann D. F. Sørensen M. A. Svenningsen S. L. (2022). Pitfalls in quantification of tRNA from starved *E. coli* cultures exposed by validation of RNA purification methods. Manuscript submitted for publication. 10.1128/mbio.02805-22 PMC9973347 36598190

[ref35] Rinas U. Hellmutii K. Kang R. Seeger A. Schlieker H. (1995). Entry of *Escherichia coli* into stationary phase is indicated by endogenous and exogenous accumulation of nucleobases. Appl. Environ. Microbiol. 61, 4147–4151. doi: 10.1128/aem.61.12.4147-4151.1995, PMID: 8534082 PMC167726

[ref36] Ross W. Sanchez-Vazquez P. Chen A. Y. Lee J. H. Burgos H. L. Gourse R. L. (2016). ppGpp binding to a site at the RNAP-DksA Interface accounts for its dramatic effects on transcription initiation during the stringent response. Mol. Cell 62, 811–823. doi: 10.1016/j.molcel.2016.04.029, PMID: 27237053 PMC4912440

[ref37] Ross W. Vrentas C. E. Sanchez-Vazquez P. Gaal T. Gourse R. L. (2013). The magic spot: a ppGpp binding site on *E. coli* RNA polymerase responsible for regulation of transcription initiation. Mol. Cell 50, 420–429. doi: 10.1016/j.molcel.2013.03.021, PMID: 23623682 PMC3654024

[ref38] Scott M. Gunderson C. W. Mateescu E. M. Zhang Z. Hwa T. (2010). Interdependence of cell growth and gene expression: origins and consequences. Science (1979) 330, 1099–1102. 10.1126/science.1192588 21097934

[ref39] Scott M. Hwa T. (2011). Bacterial growth laws and their applications. Curr. Opin. Biotechnol. 22, 559–565. doi: 10.1016/j.copbio.2011.04.014 21592775 PMC3152618

[ref40] Scott M. Klumpp S. Mateescu E. M. Hwa T. (2014). Emergence of robust growth laws from optimal regulation of ribosome synthesis. Mol. Syst. Biol. 10:747. doi: 10.15252/msb.20145379, PMID: 25149558 PMC4299513

[ref41] Sørensen M. A. Jensen K. F. Pedersen S. (1994). High concentrations of ppGpp decrease the RNA chain growth rate. Implications for protein synthesis and translational fidelity during amino acid starvation in *Escherichia coli*. J. Mol. Biol. 236, 441–454. doi: 10.1006/jmbi.1994.1156, PMID: 7508988

[ref42] Sørensen M. A. Pedersen S. (1998). Determination of the peptide elongation rate in vivo. Methods Mol. Biol. 77, 129–142. 9770666 10.1385/0-89603-397-X:129

[ref43] Spira B. Silberstein N. Yagil E. (1995). Guanosine 3′,5′-bispyrophosphate (ppGpp) synthesis in cells of *Escherichia coli* starved for P(i). J. Bacteriol. 177, 4053–4058. doi: 10.1128/jb.177.14.4053-4058.1995, PMID: 7608079 PMC177136

[ref44] Stenum T. S. Sørensen M. A. Svenningsen S. L. (2017). Quantification of the abundance and charging levels of transfer RNAs in *Escherichia coli*. J. Vis. Exp. 126, 1–10. doi: 10.3791%2F56212 10.3791/56212 PMC5614356 28872118

[ref45] Sulthana S. Basturea G. N. Deutscher M. P. (2016). Elucidation of pathways of ribosomal RNA degradation: an essential role for RNase E. RNA 22, 1163–1171. doi: 10.1261/rna.056275.116, PMID: 27298395 PMC4931109

[ref46] Vanbogelen R. A. Olson E. R. Wanner B. L. Neidhardt F. C. (1996). Global analysis of proteins synthesized during phosphorus restriction in *Escherichia coli*. J. Bacteriol. 178, 4344–4366. doi: 10.1128/jb.178.15.4344-4366.1996, PMID: 8755861 PMC178200

[ref47] Vind J. Sørensen M. A. Rasmussen M. D. Pedersen S. (1993). Synthesis of proteins in *Escherichia coli* is limited by the concentration of free ribosomes. Expression from reporter genes does not always reflect functional mRNA levels. J. Mol. Biol. 231, 678–688. doi: 10.1006/jmbi.1993.1319, PMID: 7685825

[ref48] Wade H. E. (1952). Variation in the phosphorus content of *Escherichia coli* during cultivation. J. Gen. Microbiol. 7, 24–30. doi: 10.1099/00221287-7-1-2-24, PMID: 13011252

[ref49] Wang B. Grant R. A. Laub M. T. (2020). ppGpp coordinates nucleotide and amino-acid synthesis in *E. coli* during starvation. Mol. Cell 80, 29–42.e10. doi: 10.1016/j.molcel.2020.08.005, PMID: 32857952 PMC8362273

[ref50] Wanner B. L. (1996). “Phosphorus assimilation and control of the phosphate regulon” in *Escherichia coli* and Salmonella Typhimurium: Cellular and Molecular Biology. ed. Neidhardt F. C. (American Society for Microbiology Press: Washington, DC), 1357–1381.

[ref51] Winther K. S. Roghanian M. Gerdes K. (2018). Activation of the stringent response by loading of RelA-tRNA complexes at the ribosomal A-site. Mol. Cell 70, 95–105.e4. doi: 10.1016/j.molcel.2018.02.033, PMID: 29625042

[ref52] Zundel M. A. Basturea G. N. Deutscher M. P. (2009). Initiation of ribosome degradation during starvation in *Escherichia coli*. RNA 15, 977–983. doi: 10.1261/rna.1381309, PMID: 19324965 PMC2673067

