# Supplementary material for: Escherichia coli protein synthesis is limited by mRNA availability rather than ribosomal capacity during phosphate starvation
Source: Front Microbiol. 2022 Dec 22;13:989818. doi: 10.3389/fmicb.2022.989818 (PMC9814008; doi:10.3389/fmicb.2022.989818)
Supplement: Supplementary file 1 [file Data_Sheet_1.PDF]

Supplementary information for

Escherichia coli protein synthesis is limited by mRNA availability rather than ribosomal capacity during phosphate starvation

Rocio Espinosa[1], Michael Askvad Sørensen[1*], Sine Lo Svenningsen[1*]

[1]Section for Biomolecular Sciences, Department of Biology, University of Copenhagen, Ole Maaløes Vej 5, 2200 Copenhagen, Denmark

* Correspondence: Address correspondence to Michael Askvad Sørensen: mas@bio.ku.dk and Sine Lo Svenningsen: sls@bio.ku.dk.

List of supplementary figures:

Supplementary Figure S1. Macromolecular profile of E. coli MAS1190 during balanced growth and after filtration into P-free medium. The figure relates to Figure 1 of the main text.

Supplementary Figure S2. Accumulation of 35S-methionine in protein during balanced growth and after induction of the *rpsA'*-*'lacZ* hybrid mRNA in *E. coli* MAS1143/pIV18. The figure relates to Figure 2B of the main text.

Supplementary Figure S3. Autoradiograms of 2D-gels showing ^{35}S-methionine pulse labeled proteins from MAS1143/pIV18 expressed under phosphate starvation in the presence or absence of IPTG. The figure relates to Figure 3 of the main text.

Supplementary Figure S4. Macromolecular profile and viability measurements of E. coli strain REP37/pIV18 with or without induction of *rpsA'*-*'lacZ* hybrid mRNA. The figure relates to Figure 4 of the main text.

Supplementary Figure S1

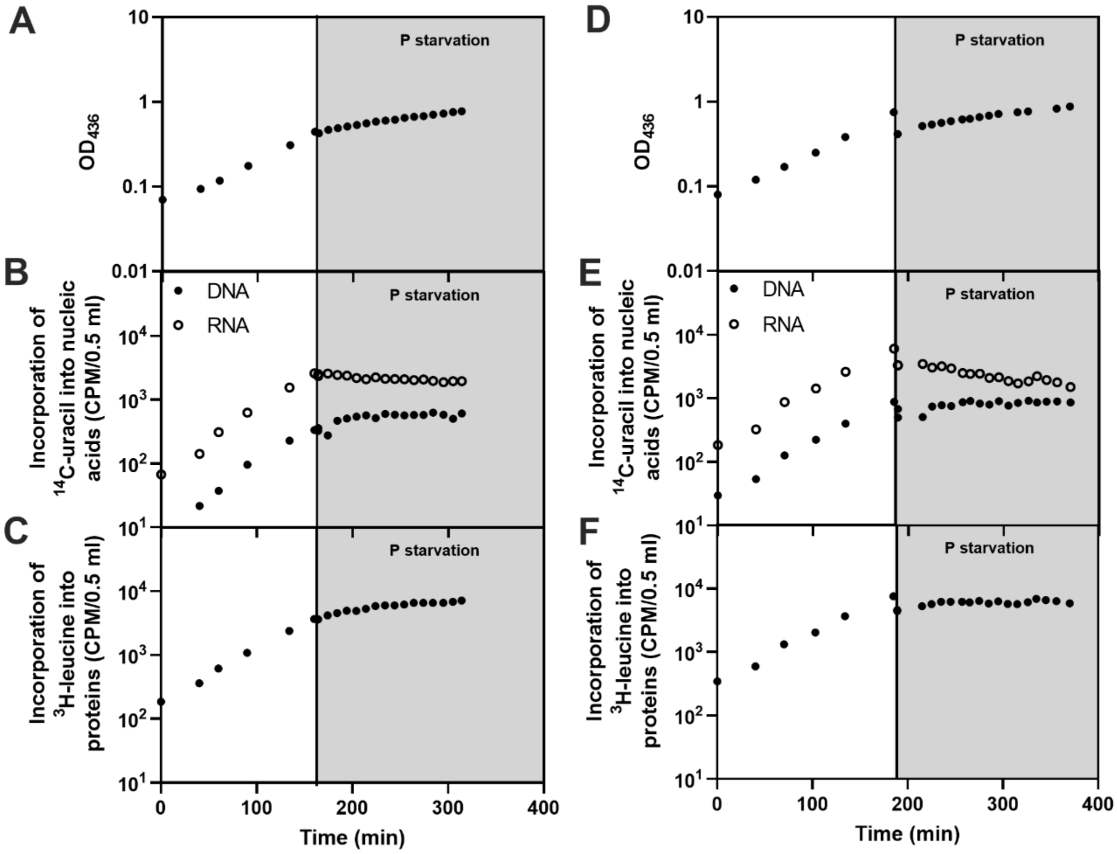

Supplementary Figure S1. Macromolecular profile of *E. coli* MAS1190 (Δ*argG* Δ*leuA* Δ*pyrE::tet*) during balanced growth (white background) and after filtration into P-free medium (gray background). The panels show **(A, D)** optical density of the culture, **(B, E)** ^{14}C-uracil incorporated into DNA (filled circles) and RNA (open circles), and **(C, F)** ^{3}H-leucine incorporated into protein. The data set presented in this figure are two independent biological replicates (A-C, and D-F) of the data presented in Figure 1 in the main text. Cells grew with a doubling time of approximately 57 minutes prior to starvation. The minor drop observed in all measurements immediately upon starvation was caused by the loss of cells by filtration. Note the logarithmic scale on the Y-axes.

Supplementary Figure S2

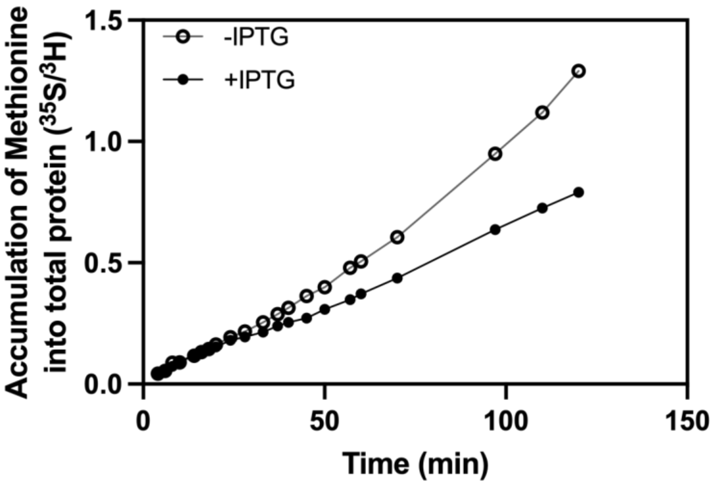

Supplementary Figure S2. Accumulation of ^{35}S-methionine into protein during balanced growth (open circles) and after induction of the *rpsA'*-'*lacZ* mRNA with IPTG (closed circles) in *E. coli* strain MAS1143 with pIV18. The ratio of $^{35}S/^{3}H$ counts is represented over time. At time "0", ^{35}S-methionine was added to a culture in balanced growth that was pre-labelled with ^{3}H-lysine. The culture was divided into two flasks, and IPTG was added to one of them at time +9 min. The data presented in this figure is an independent biological replicate of Figure 2B in the main text.

Supplementary Figure S3

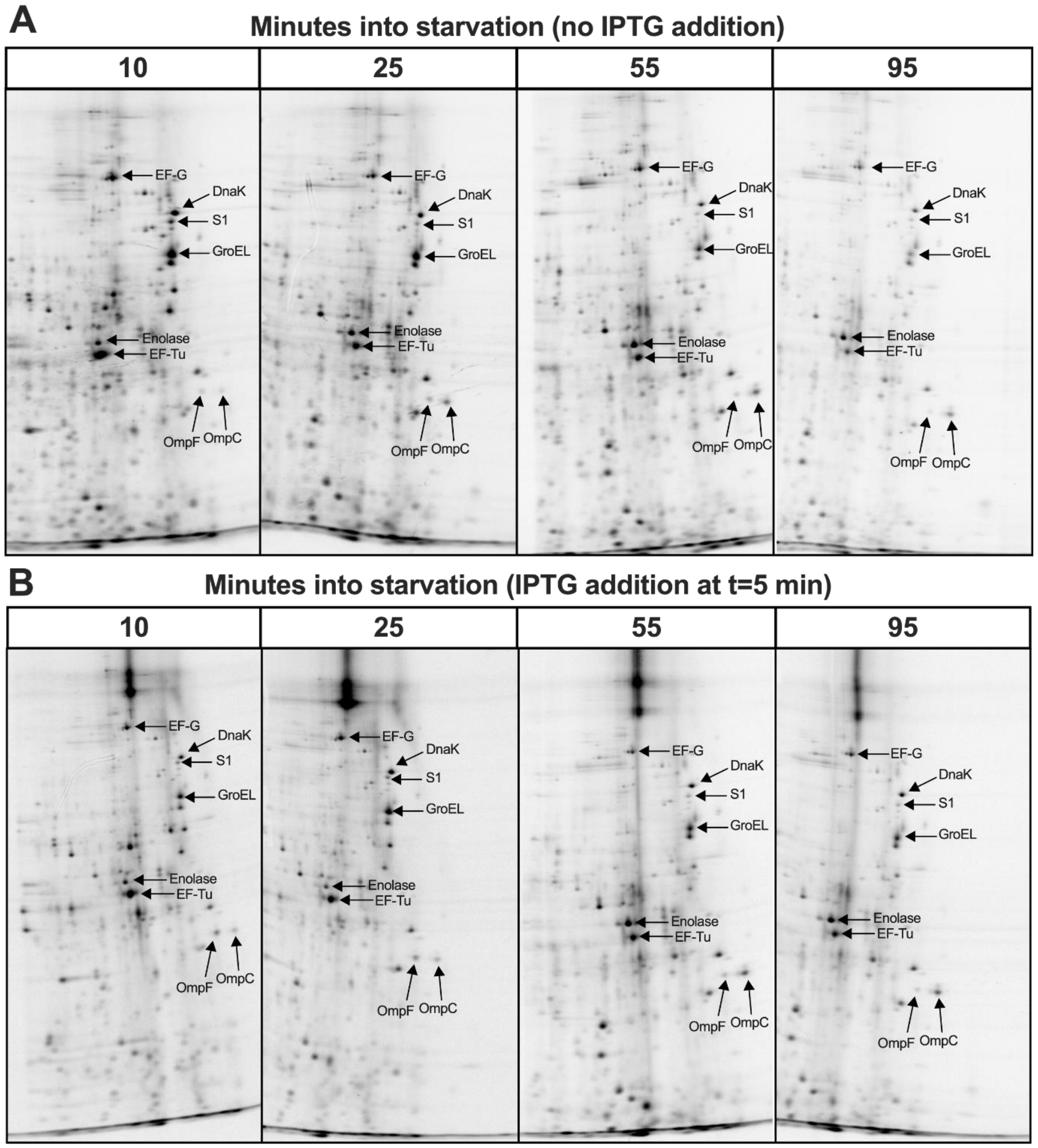

Supplementary Figure S3. Autoradiograms of 2D-gels showing ^{35}S-methionine pulse labeled proteins expressed under phosphate starvation. Proteins were focused to their charge equilibrium in the first dimension (acidic side left) and next separated by molecular weight in the second dimension on a 10.5% SDS-PAGE gel (descending MW). **A)** Expression pattern of *E. coli* MAS1143/pIV18 proteins from pulse labelling after P starvation at the time points indicated. Arrows with names point to identified protein spots for orientation. **B)** As panel A, except IPTG was added to the culture at time +5 min.

Supplementary Figure S4

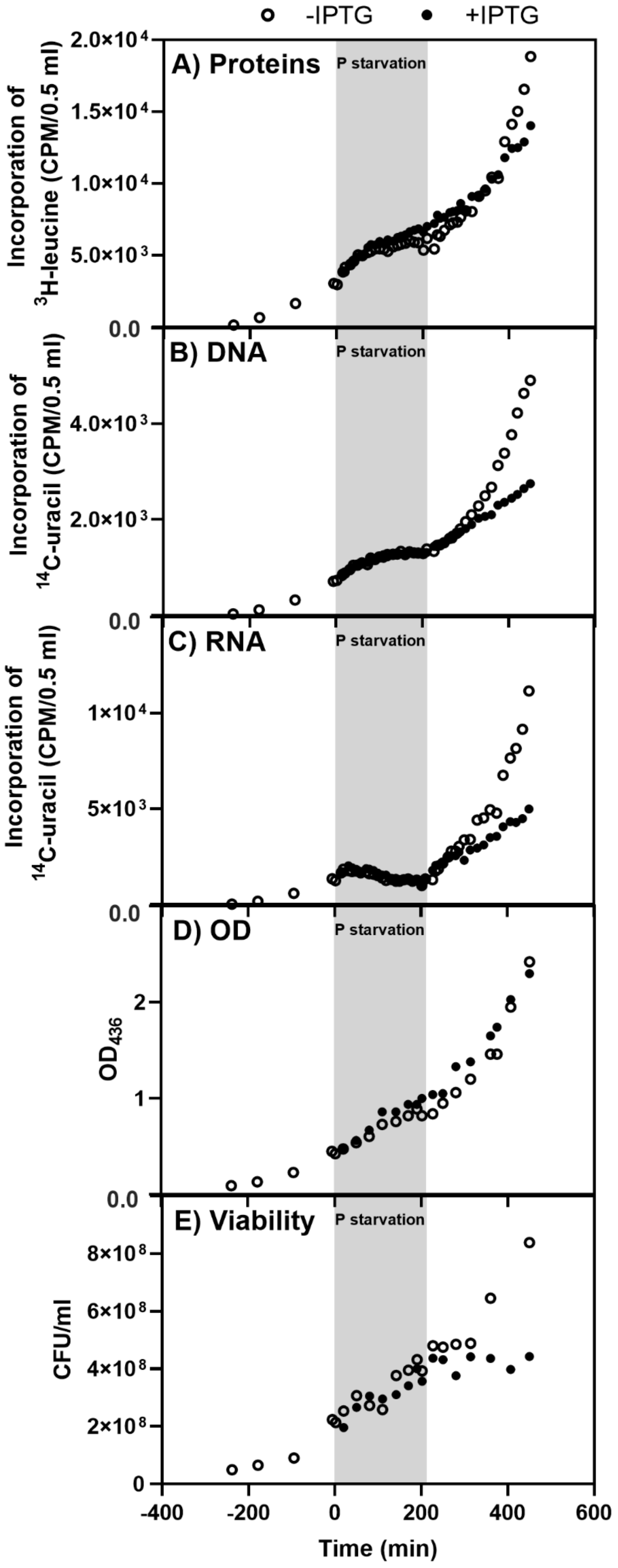

Supplementary Figure S4. Macromolecular profile of *E. coli* strain REP37/pIV18 *(ΔargG ΔleuA ΔpyrE::tet lacI^{q1} lacZ::Tn5*) with or without induction of long-lived hybrid mRNA during P starvation (gray background) and refeed (white background). The panels show accumulation of **A)** proteins, **B)** DNA, **C)** RNA, and also **D)** optical density,

and **E)** cell viability as CFU/ml. The data shown in this figure is an independent biological replicate of Figure 4 in the main text. Note the linear scale on the Y-axes.
